# Paracentesis outcomes from a medicine procedure service at a tertiary care transplant center

**DOI:** 10.1002/jhm.70037

**Published:** 2025-03-27

**Authors:** Pete Meliagros, Benjamin Chopski, Matthew Ambrosio, Stanley Liu, Somaya Albhaisi, Lana Petrova, Evan Ritter, Adam Garber

**Affiliations:** ^1^ Department of Internal Medicine, Hospital Medicine Virginia Commonwealth University Health System/VCU Health Richmond Virginia USA; ^2^ Division of Hospital Medicine, Department of Internal Medicine Virginia Commonwealth University Health System/VCU Health Richmond Virginia USA; ^3^ Department of Biostatistics Virginia Commonwealth University Richmond Virginia USA; ^4^ Virginia Commonwealth University School of Medicine Richmond Virginia USA; ^5^ Department of Internal Medicine Virginia Commonwealth University Health System/VCU Health Richmond Virginia USA

## Abstract

**Background:**

Paracentesis is a commonly performed procedure with overall low complication rates. There is a paucity of modern data investigating outcomes for inpatients using standardized point of care ultrasound.

**Objectives:**

We aimed to evaluate complication rates and outcomes of paracentesis in patients in the inpatient setting of a large tertiary transplantation center.

**Methods:**

We identified patients with ascites of multiple etiologies undergoing paracentesis by a medicine procedure service at a university center. Univariate and multivariate analyses were conducted to identify clinical and demographic factors associated with kidney injury (AKI) or significant HGB drop (≥2 g/dL).

**Results:**

Of 1746 patients, 12% of patients receiving small volume (≤5 L) and 10% receiving large volume paracentesis (>5 L) developed a post procedural AKI (OR 0.857, 95% CI: 0.633–1.154) with no significant difference between groups (*p* = .30). In multivariable analysis, Model for End‐Stage Liver Disease Sodium (MELD‐Na) score as a continuous variable had a more significant impact in the development of AKI (OR 1.15 CI 1.08–1.22, *p* < .001) as well as patients who were noted to be receiving paracentesis due to clinical deterioration (OR 2.48 CI 1.08–5.7, *p* < .03). Of 2034 patients, 94% of patients had no significant drop in hemoglobin (<2 g/dL). There was no significant difference in BMI (12.73 vs. 16.68, *p* = .6), INR (1.6 vs. 1.6, *p* = .8), or platelet count (114 vs. 106, *p* > .9) between groups.

**Conclusion:**

Regardless of volume of ascites removed, paracentesis is associated with a low risk of AKI, however there was an increased risk in the clinically decompensating patient. The bleeding risk was also found to be low when performed by experienced proceduralists on a procedure service.

## INTRODUCTION

Bedside paracentesis is a commonly performed procedure across various patient care settings. Overall, this is considered a safe procedure with a low likelihood of complication rates, estimated to be less than 2%.^1^ Given the low prevalence of complications, outcomes data from different settings is often combined to reach statistical power to detect factors contributing to adverse outcomes or complications. This could miss certain clinical factors unique to hospitalized patients.

Additionally, some outcomes data from older studies were published before current standards regarding IV albumin infusion or point‐of‐care ultrasound coalesced.^1^, ^2^ Few studies investigate the experience of primary operator, which could also have an effect on procedural risk.^3^, ^4^ Furthermore, outcomes data also precede the adoption of medicine procedure services (MPS).

While complication rates from paracenteses are low, it can be fatal or life‐threatening, requiring additional procedures.^1^ A systematic review noted 40% of hemorrhagic complications required a surgical (35%) or IR (65%) intervention, with mortality rates as high as 43% from paracentesis‐associated hemorrhage.^5^, ^6^ Additionally, some complications can be days to a week after paracentesis in rare instances, which may lead to missed detection and underestimate true complication rates.^7^, ^8^

We aimed to evaluate complication rates and outcomes of paracentesis in patients in the inpatient setting of a large tertiary transplantation center. We included patients with decompensated cirrhosis with high MELD scores that are at a perceived higher risk of procedural complications. We also included patients with other reasons for ascites, including cardiogenic ascites, which is not captured in prior literature. Additionally, we evaluated operator outcomes data from our MPS, consisting of residents and a supervising hospitalist attending, who perform bedside procedures utilizing a standardized technique.

## METHODS

### Setting

Virginia Commonwealth University (VCU) Health is an 820‐bed tertiary care liver‐kidney transplant center. The VCU MPS is a consultation service consisting of a hospitalist attending and 0–2 internal medicine residents, staffed 7 days a week. Consults are provided to all medical and surgical service lines. Procedures are performed at the bedside by residents under direct supervision, or by the proceduralist if no resident is available. Static site marking is completed via 2‐probe technique using phased (or curvilinear) and linear arrays to investigate pocket size and subcutaneous vessels as this has been found to reduce bleeding risk.^9^ Paracentesis is completed using an 8 Fr abdominal drainage catheter (Teleflex Inc). 18‐ or 20‐gauge standard angiocaths may be used for diagnostic paracentesis, certain small‐volume procedures, or procedures deemed to be higher risk. IV Albumin infusion was administered postprocedure with large volume paracentesis (LVP) per the American Association for the Study of Liver Disease (AASLD) guidelines.

### Study design and population

We conducted a prospective cohort study from March 2017 to January 2023 of inpatient paracentesis procedures performed by a MPS on hospitalized patients outside of the medical intensive care unit with ascites. Patients were followed during the study period and demographic and clinical data were collected in a Research Electronic Data Capture (REDCap) database in a registry format.^10^, ^11^ The total cohort included 2651 individuals.

Exclusion criteria were applied separately to each sub‐cohort to maximize available data. 905 patients were excluded in the acute kidney injury sub‐cohort due to hemodialysis status or incomplete data. 618 were excluded from complications and mortality analysis due to incomplete data. 617 were excluded from the hemoglobin (HGB) analysis due to incomplete data. The study protocol was approved as exempt by our institutional IRB.

### Outcome measures

Manual chart review was performed to obtain additional retrospective data not captured in the prospective database. Demographic data collected included age, gender, race/ethnicity, and body mass index (BMI). Descriptive data about the procedure included indication for paracentesis; etiology of ascites; whether the procedure was therapeutic and/or diagnostic; operator and supervisor; number of attempts; site of paracentesis, depth of pocket, and amount of fluid drained (see appendix). For the paracentesis indication of “clinical deterioration,” we defined this as a change in vital signs (e.g., fever, new or worsening hypotension), altered mental status, worsening leukocytosis, and/or worsening renal function. Prior guidelines led to our use of >5 L for the definition of LVP.^12^, ^13^, ^14^ Lab studies collected were serum sodium, liver function tests (AST, ALT, total bilirubin), renal function (normal, acute kidney injury, chronic kidney disease, or requiring dialysis), serum creatinine pre‐procedure and postprocedure, HGB pre‐ and postprocedure, platelets, PT/INR, and PTT all within 48 h of procedure.

Other patient factors that were collected included whether the patient was on anticoagulation, whether it was held for the procedure, and the use of antibiotics at the time of the procedure. Both immediate and delayed complications were noted, including AKI, receiving a blood transfusion within 48 h postprocedure, and death during admission. Chart reviews were used to discern whether deaths (within 7 days of procedure) and hemorrhagic complications (HGB drop by ≥2 g/dL) were attributable to the procedure. Reviewers considered whether an imaging or diagnostic procedure was performed to confirm the source of hemorrhage, whether an alternate source of bleeding was documented, and whether the patient required ICU‐level care within 48 h postprocedure. If answers to these questions were insufficient to identify if the hemorrhage was related to the procedure, we considered whether alternate explanations of a drop in HGB (i.e., albumin infusions, hemodilution) are as likely to be the cause as the procedure. When reviewing patients in our study period for predictors of HGB drop, all researchers performing chart review followed a standardized protocol that was created to ensure consistency in data collection.

### Statistical analysis

We performed two separate analyses using R version 4.3.1 (Vienna, Austria). Separate exclusion criteria were used for each analysis to maximize sample size for each.

The first analysis, with the aim to assess the effect of LVP on postprocedure AKI, baseline characteristics, and outcomes were summarized, stratified by large versus small volume of fluid removed during paracentesis to compare demographics between groups. Continuous variables were displayed as median (range) and compared between groups using a Wilcoxon rank sum test, while categorical variables were displayed as *n* (%) and compared between groups using Pearson's Chi‐squared test. The main outcome of interest, postprocedure AKI, was defined as any of the following: a drop in eGFR of >50% after the procedure (calculated using the 2021 CKD‐EPI Creatinine formula for eGFR), an increase in creatinine of >50% after the procedure, or an increase in creatinine of ≥0.3 mg/dL after the procedure.

In addition to univariate analysis, a multivariable mixed effects logistic regression model was used to assess predictors of post‐paracentesis AKI, adjusting for the random effect of the individual receiving paracentesis, as some individuals received multiple procedures. Due to the presence of many potential covariates, we first ran a univariate mixed effect logistic regression model on post‐paracentesis AKI with every baseline covariate defined in Table 1 as the predictor variable, and only selected covariates with significant individual association with post‐paracentesis AKI. Volume of fluid removed (as a binary variable) and age were then included with all the individually significant covariates in a multivariable mixed effects model, and backward stepwise regression was performed to determine the set of covariates that best predict post‐paracentesis AKI and to determine if the volume of fluid removed was a significant predictor. We also plotted volume removed against change in eGFR postprocedure as continuous variables to display the relationship between the variables on a continuous scale.

**Table 1:** Baseline characteristics of small versus large volume paracentesis.

Characteristic	Overall, *N* = 1746^a^	Small volume, *N* = 955^a^	Large volume, *N* = 791^a^	*p* Value^b^
Age	58.00 (19.00, 90.00)	58.00 (19.00, 90.00)	59.00 (25.00, 85.00)	.11
Sex				<.001
Female	608 (35%)	390 (41%)	218 (28%)	
Male	1138 (65%)	565 (59%)	573 (72%)	
Race				<.001
Asian	20 (1.2%)	16 (1.7%)	4 (0.5%)	
Black	447 (26%)	267 (28%)	180 (23%)	
Hispanic	41 (2.4%)	26 (2.7%)	15 (1.9%)	
Other	22 (1.3%)	17 (1.8%)	5 (0.6%)	
White	1208 (70%)	626 (66%)	582 (74%)	
CKD	953 (55%)	517 (54%)	436 (55%)	.7
AKI only	595 (34%)	348 (36%)	247 (31%)	.022
Serum creatinine	1.29 (0.20, 12.84)	1.29 (0.20, 12.84)	1.30 (0.23, 9.17)	.5
Serum sodium	134.00 (115.00, 153.00)	134.00 (115.00, 151.00)	134.00 (116.00, 153.00)	.6
Total bilirubin	2.40 (0.05, 58.10)	2.50 (0.05, 58.10)	2.40 (0.20, 57.10)	.4
INR	1.60 (0.90, 211.00)	1.60 (0.90, 211.00)	1.60 (0.90, 71.60)	.6
INR group				.2
<2	1,227 (74%)	654 (73%)	573 (75%)	
≥2	437 (26%)	248 (27%)	189 (25%)	
Platelet count	116.00 (1.80, 1115.00)	118.00 (1.80, 1000.00)	110.00 (23.00, 1115.00)	.044
PLT group				>.9
≤50	184 (11%)	100 (11%)	84 (11%)	
>50	1552 (89%)	847 (89%)	705 (89%)	
RBC fluid	513.00 (0.00, 900,000.00)	654.00 (0.00, 900,000.00)	382.00 (0.00, 851,566.00)	.003
Anticoagulation				.8
None	1245 (76%)	673 (76%)	572 (76%)	
Prophylactic	289 (18%)	154 (17%)	135 (18%)	
Therapeutic	104 (6.3%)	59 (6.7%)	45 (6.0%)	
MELD‐NA score	23.00 (6.00, 40.00)	23.00 (6.00, 40.00)	23.00 (7.00, 40.00)	.7
BMI	27.13 (12.73, 84.00)	26.59 (13.00, 84.00)	28.00 (12.73, 71.30)	.002
Multiple attempts	179 (10%)	120 (13%)	59 (7.5%)	<.001
Depth of pocket	6.00 (2.00, 16.00)	5.00 (2.00, 15.00)	6.00 (3.00, 16.00)	<.001
AST	57.00 (9.00, 797.00)	60.00 (9.00, 637.00)	55.00 (9.00, 797.00)	.080
ALT	24.00 (5.00, 717.00)	25.00 (5.00, 507.00)	23.00 (5.00, 717.00)	.14
Previous paracentesis procedures	0.00 (0.00, 21.00)	0.00 (0.00, 21.00)	1.00 (0.00, 19.00)	<.001
Amount of volume removed	4.86 (0.00, 26.00)	3.40 (0.00, 5.00)	6.90 (5.05, 26.00)	<.001
Antibiotics	287 (46%)	172 (48%)	115 (43%)	.2
eGFR before paracentesis	57.63 (3.98, 182.39)	57.33 (3.98, 168.33)	58.63 (5.62, 182.39)	>.9
Performed by				.3
Attending	357 (20%)	204 (21%)	153 (19%)	
Resident	1389 (80%)	751 (79%)	638 (81%)	
Death within 7 days	10 (0.6%)	8 (0.8%)	2 (0.3%)	.12
Etiology				
Alcoholic liver disease	826 (47%)	426 (45%)	400 (51%)	.013
Autoimmune	26 (1.5%)	11 (1.2%)	15 (1.9%)	.2
Cardiogenic	107 (6.1%)	52 (5.4%)	55 (7.0%)	.2
Chronic viral hepatitis	295 (17%)	145 (15%)	150 (19%)	.036
Malignant	160 (9.2%)	119 (12%)	41 (5.2%)	<.001
Metabolic‐dysfunction associated steatotic liver disease	232 (13%)	109 (11%)	123 (16%)	.011
Multiple causes	168 (9.6%)	77 (8.1%)	91 (12%)	.015
Other etiology	86 (4.9%)	48 (5.0%)	38 (4.8%)	.8
Unknown etiology	177 (10%)	117 (12%)	60 (7.6%)	.001
Indication				
New onset ascites	237 (14%)	150 (16%)	87 (11%)	.004
Clinical deterioration of patient with ascites	239 (14%)	161 (17%)	78 (9.9%)	<.001
Management of tense/diuretic resistant ascites	1323 (76%)	671 (70%)	652 (82%)	<.001
Other	11 (0.6%)	9 (0.9%)	2 (0.3%)	.13
Outcomes				
Post Paracentesis AKI	198 (11%)	115 (12%)	83 (10%)	.3
Transfusion postprocedure	76 (12%)	48 (13%)	28 (11%)	.3
Death within 7 days	10 (0.6%)	8 (0.8%)	2 (0.3%)	.12

*Note*: Displays baseline demographics and outcomes, compared between large volume and small volume paracentesis. Large volume paracentesis is defined as >5 L removed during procedure.

^a^ Median (Range); *n* (%).

^b^ Wilcoxon rank sum test; Pearson's Chi‐squared test; Fisher's exact test.

The second analysis, aimed to identify predictors of a drop in HGB after paracentesis, used similar statistical methods. Baseline characteristics were summarized, stratified by HGB drop versus no HGB drop, and tested for statistical difference between the groups using the same methods. The same multivariable backwards stepwise method to create a mixed effects regression model was used to identify significant predictors of HGB drop, however a linear regression model was used as opposed to a logistic regression model, so the outcome was defined as drop in HGB g/dl as a continuous variable. Extreme values of change in HGB (>10 g/dL) were excluded from linear regression as outliers. Linear regression was chosen instead of logistic regression because only 117 (5.8%) of patients had a significant HGB drop to avoid biasing the results in a logistic regression. Volume of fluid removed was also added to the multivariable regression to determine its effect on HGB drop.

## RESULTS

A total of 2651 patients had a bedside paracentesis performed by our MPS over our study time period. After applying exclusion criteria, 1746 patients were included in our analysis (826 were excluded for missing data points, and 79 were on hemodialysis). Table 1 displays the baseline characteristics and outcomes for the study cohort on LVP. 953 (55%) patients had chronic kidney disease (CKD) with a median creatinine of 1.29 mg/dL. The most common etiology of cirrhosis was alcohol‐induced (EtOH) (47%), followed by hepatic C virus (17%). Median MELD‐Na score was 23. 955 (55%) patients underwent small volume, and 791 (45%) patients underwent LVP. There was no significant difference found in the development of AKI postprocedure between the groups: 115 (12%) vs. 83 (10%) in the small vs. large volume group respectively developed AKI (*p* = .3).

**Figure 1:**
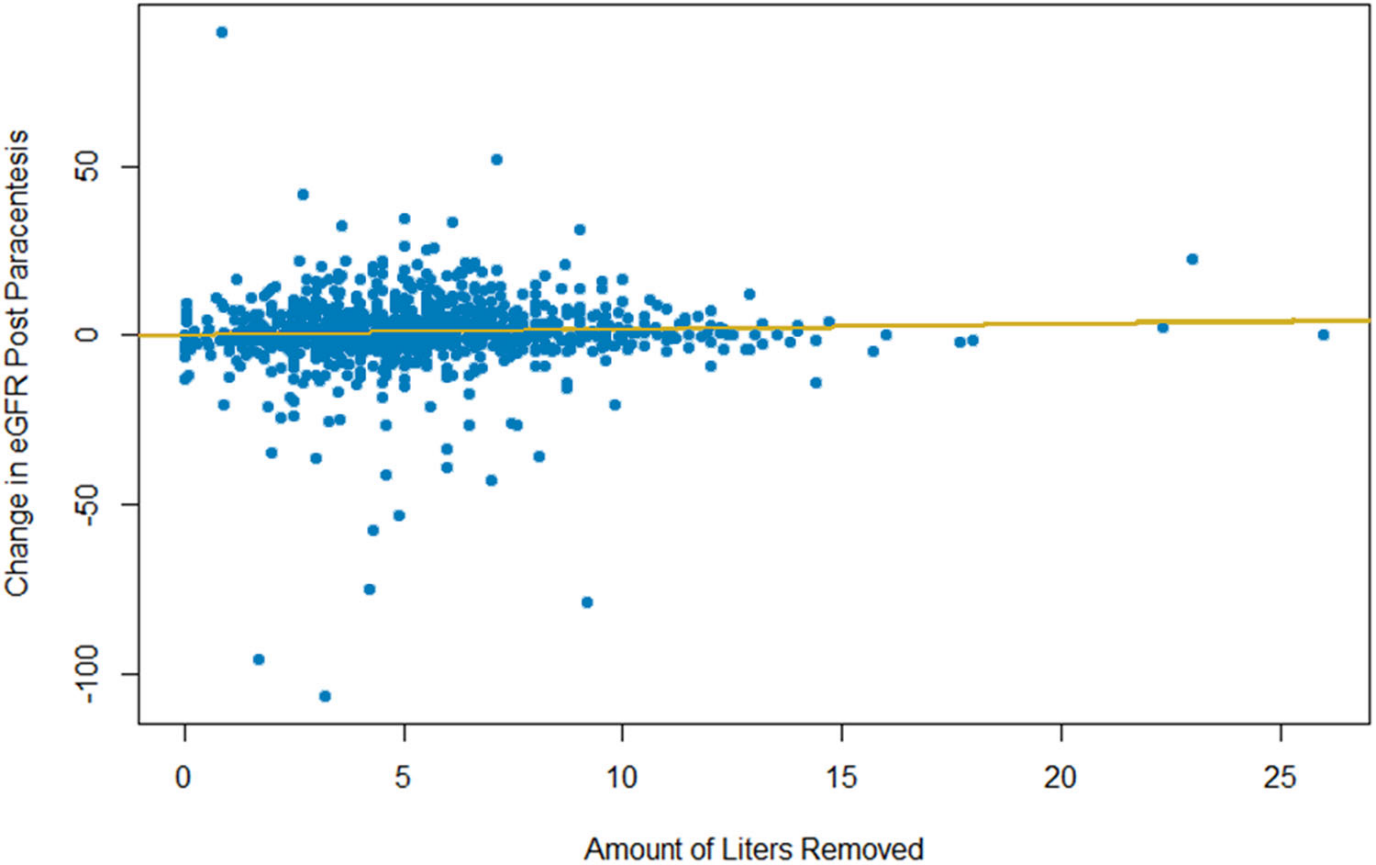
Association between volume removed during paracentesis and drop in eGFR post‐paracentesis. Plots the association of liters removed during the procedure and change in eGFR postoperation on a continuous scale. There appears to be no trend between the two variables.

Table 2a shows the results of the multivariable stepwise logistic regression on post‐paracentesis AKI. The only significant predictors found in the model were MELD‐Na score and clinical deterioration as the indication for paracentesis. For every 1 pt increase in MELD‐Na score, the risk of AKI increased by 15%. Importantly, volume of paracentesis was not a significant predictor in this model. Our findings suggest that LVP does not have an association with developing AKI after paracentesis, whether performed as a univariate analysis or adjusted for other covariates. Figure 1 plots the amount of volume removed against change in eGFR as continuous variables. There is no trend in the plot to indicate an association of LVP with postprocedure AKI.

**Table 2:** Stepwise multivariate logistic regression.^a^

2a: Post‐paracentesis AKI			
Predictors	Odds ratios	CI	*p*
MELD NA Score	1.15	1.08–1.22	<0.001
Clinical deterioration	2.49	1.08–5.72	0.032
Age	0.99	0.95–1.04	0.721

2b: Change in HGB postprocedure			
Predictors	Estimates	CI	*p*
Large volume paracentesis [>5]	−0.19	−0.30 to −0.08	0.001
AST	−0.0010	−0.0017 to −0.0003	0.001
Depth of Pocket	−0.03	−0.06 to −0.01	0.018

*Note* (2a): Baseline covariates with significant univariate association with AKI were initially included in the model, in addition to age and volume removed, and this table displays the final model after variable selection. The only variables with significant association with postprocedure AKI when adjusted for other covariates were MELD score and clinical deterioration as etiology. Volume of paracentesis was not a significant predictor.

*Note* (2b): HGB drop postprocedure served as the continuous outcome variable. Demographic variables with significant univariate association with HGB drop were included in the model, and this figure displays the final selected model after backwards stepwise variable selection. When adjusted for other variables, depth of pocket, AST, and volume removed were the significant predictors identified. All of these variables have a negative association with change in HGB, indicating that as they increase, we expect HGB to drop postprocedure.

a Displays mixed effect stepwise multivariate logistic regression, adjusted for the random effect of repeated procedures on the same patient.

Table 3 displays the baseline characteristics and outcomes for the analysis to identify predictors of HGB drop after paracentesis. Of the 2034 patients included in this cohort, 94% of patients had no significant drop in their HGB (<2 g/dL). 117 patients met the inclusion criteria of a significant HGB drop (>2 g/dL) and upon chart review, only 10 patients (0.49%) were confirmed to have bleeding secondary to paracentesis. There was no significant difference in BMI (median of 12.73 vs. 16.68, *p* = .6), median INR (median 1.60 vs. 1.60, *p* = .8), or platelet count (median of 114 vs. 106, *p* > .9) between groups. A similar proportion of patients were on DVT prophylaxis or therapeutic anticoagulation in both groups (17% vs. 14% and 6.1% vs. 4.5%, respectively, *p* = .4). Patients with chronic viral hepatitis were more likely to have a significant HGB drop (*p* = .03). There was no significant difference between diagnostic vs. therapeutic attempts or whether an attending or resident performed the procedure. 14% of patients with HGB drop of <2 g/dL received blood transfusion, while 30% received a transfusion if significant HGB drop (*p* < .004). Of note, there was a significant difference (*p* < .01) in the number of individuals deceased within 7 days after the procedure: 3.4% versus 0.6% of patients with significant drop in HGB, but upon further chart review only 1.7% were found to be related to the procedure (*p* = .13).

**Table 3:** Baseline characteristics stratified by HGB drop.

Characteristic	Overall, *N* = 2034^a^	No HGB drop ≥2, *N* = 1917^a^	HGB drop ≥2, *N* = 117^a^	*p* Value^b^
HGB drop	−0.30 (−5.90, 68.80)	−0.20 (−1.90, 68.80)	−2.40 (−5.90, −2.00)	<.001
Age	58.00 (19.00, 90.00)	58.00 (19.00, 90.00)	58.00 (26.00, 82.00)	.8
Race				.5
Asian	20 (1.0%)	19 (1.0%)	1 (0.9%)	
Black	521 (26%)	494 (26%)	27 (23%)	
Hispanic	51 (2.5%)	46 (2.4%)	5 (4.3%)	
Other	25 (1.2%)	25 (1.3%)	0 (0%)	
White	1408 (70%)	1324 (69%)	84 (72%)	
Sex				.3
Female	745 (37%)	707 (37%)	38 (32%)	
Male	1289 (63%)	1210 (63%)	79 (68%)	
BMI	27.00 (12.73, 84.00)	27.00 (12.73, 84.00)	27.79 (16.68, 50.00)	.6
RBC fluid	596.00 (0.00, 900,000.00)	617.00 (0.00, 900,000.00)	300.00 (1.00, 900,000.00)	.025
INR	1.60 (0.90, 211.00)	1.60 (0.90, 211.00)	1.60 (1.00, 5.80)	.8
INR group				.7
<2	1386 (72%)	1302 (71%)	84 (73%)	
≥2	552 (28%)	521 (29%)	31 (27%)	
Platelet count	113.00 (0.75, 1115.00)	114.00 (0.75, 1115.00)	106.00 (9.00, 552.00)	>.9
PLT group				.071
≤50	226 (11%)	219 (11%)	7 (6.0%)	
>50	1800 (89%)	1691 (89%)	109 (94%)	
Anticoagulation				.4
None	1482 (77%)	1391 (76%)	91 (82%)	
Prophylactic	333 (17%)	318 (17%)	15 (14%)	
Therapeutic	116 (6.0%)	111 (6.1%)	5 (4.5%)	
eGFR	58.38 (3.98, 182.39)	57.00 (3.98, 182.39)	79.36 (8.55, 133.09)	.016
MELD‐NA score	24.00 (6.00, 40.00)	24.00 (6.00, 40.00)	23.00 (9.00, 40.00)	.3
Multiple attempts	216 (11%)	207 (11%)	9 (7.8%)	.3
Depth of pocket	5.00 (1.00, 16.00)	5.00 (1.00, 16.00)	5.00 (2.00, 16.00)	.086
AST	59.00 (7.00, 2776.00)	57.00 (7.00, 2746.00)	81.00 (23.00, 2776.00)	<.001
ALT	25.00 (5.00, 947.00)	24.00 (5.00, 947.00)	35.00 (5.00, 476.00)	<.001
Previous paracentesis procedures	0.00 (0.00, 21.00)	0.00 (0.00, 21.00)	0.00 (0.00, 17.00)	.10
Therapeutic/diagnostic				.14
Both therapeutic/diagnostic	1117 (55%)	1045 (55%)	72 (62%)	
Diagnostic only	372 (18%)	348 (18%)	24 (21%)	
Neither	1 (<0.1%)	1 (<0.1%)	0 (0%)	
Therapeutic only	539 (27%)	518 (27%)	21 (18%)	
Performed by				.2
Attending	432 (21%)	413 (22%)	19 (16%)	
Resident	1602 (79%)	1504 (78%)	98 (84%)	
Etiology				
Alcoholic liver disease	969 (48%)	905 (47%)	64 (55%)	.12
Autoimmune	33 (1.6%)	30 (1.6%)	3 (2.6%)	.4
Cardiogenic	103 (5.1%)	103 (5.4%)	0 (0%)	.010
Chronic viral hepatitis	326 (16%)	299 (16%)	27 (23%)	.032
Malignant	166 (8.2%)	158 (8.2%)	8 (6.8%)	.6
Metabolic‐dysfunction associated steatotic liver disease	270 (13%)	256 (13%)	14 (12%)	.7
Multiple causes	175 (8.6%)	162 (8.5%)	13 (11%)	.3
Other etiology	109 (5.4%)	107 (5.6%)	2 (1.7%)	.071
Unknown etiology	230 (11%)	218 (11%)	12 (10%)	.7
Indication				
New onset ascites	296 (15%)	271 (14%)	25 (21%)	.031
Clinical deterioration of patient with ascites	463 (23%)	432 (23%)	31 (26%)	.3
Management of tense/diuretic resistant ascites	1313 (65%)	1247 (65%)	66 (56%)	.058
Other	39 (1.9%)	39 (2.0%)	0 (0%)	.2
Volume				
Large volume paracentesis (>5 L removed)*	729 (45%)	684 (43%)	45 (49%)	.4
Outcomes				
Transfusion postprocedure	119 (15%)	105 (14%)	14 (30%)	.004
Death within 7 days	15 (0.7%)	11 (0.6%)	4 (3.4%)	.009

*Note*: Displays the univariate analysis of baseline characteristics and outcomes of those with HGB drop ≥2 g/dL compared to those without and HGB drop ≥2 g/dL. Statistical tests were used as appropriate to compare between the two groups.

^a^ Median (range); *n* (%)

^b^ Wilcoxon rank sum test; Fisher's exact test; Pearson's Chi‐squared test

* 425 patients in this cohort were missing data on Volume

The only baseline variables that had a significant difference between the HGB drop groups in univariate mixed‐effect regression analysis were: AST, ALT, INR, cardiogenic ascites, alcoholic liver disease or chronic viral hepatitis as etiology, and depth of pocket. LVP was also a significant predictor in univariate mixed effects regression, despite not having a significant difference between groups in Table 3. Utilizing these variables in mixed effects multivariable linear regression, accounting for random effect of multiple procedures performed on the same patient, we found that depth of pocket, LVP, and AST were the significant predictors identified after stepwise regression. Depth of pocket (−0.03, CI –0.06 to −0.01, *p* = .02) and AST (−0.0010, CI −0.0017 to − 0.0003, *p* = .001) and volume removed during paracentesis (−0.19, CI –0.30 to −0.08, *p* = .001) all have a negative association with change in HGB indicating that as they increase, we expect HGB to drop postprocedure (Table 2b).

## DISCUSSION

Paracentesis is a commonly performed procedure with overall risks cited around 1‐2%.^1^ We aimed to focus on risks associated with post‐paracentesis AKI and bleeding as the most studied complications.^1^ We did not investigate the incidence of other complications given low rate of occurrence (one perforation in our cohort), and we typically utilize surgical glue prophylactically to minimize ascites and anasarca leak, which would affect overall incidence.

Prior studies have shown that there is a 5%–10% risk of developing AKI with increased risk associated with higher MELD score and younger age.^15^, ^16^ Our study identified a similar risk of AKI (10%–12%) and found an association with a higher MELD‐Na score but no association with respect to age. It has previously been found that there is a 1.24x risk for each liter of fluid drained,^15^ but we did not find any correlation between volume removed and AKI. We did find that patients with clinical deterioration as an indication for paracentesis were at higher risk of developing AKI post‐paracentesis. Although we did not collect data regarding IV albumin administration, a strength of this study is that our institution strictly follows the AASLD guidelines for albumin administration, which is further reinforced by our MPS postprocedure. Our findings support that paracentesis, regardless of volume, is well tolerated by the kidneys when guideline‐recommended albumin repletion is used, but clinicians should weigh the risk of AKI against the symptomatic relief provided by therapeutic paracentesis in the clinically deteriorating patient.

Serious hemorrhagic complications of paracentesis have been seen both with LVP and diagnostic paracentesis and are categorized into three groups: abdominal wall hematoma, pseudoaneurysm, and hemoperitoneum.^17^ Mallory and Schaefer evaluated 242 diagnostic abdominal paracenteses in patients with liver disease and reported four patients with serious hemorrhagic complications (1.7%), which was significantly higher than previously published data.^18^ McVay and Toy reviewed 608 procedures in 395 patients and found the incidence of significant bleeding (defined as more than a 2 g/dL drop) was 3.1%.^19^ Delayed bleeding has also been described and could be due to rapid decompression of splanchnic circulation related to a sudden decrease in intraabdominal pressure leading to increased portal pressures and varix rupture.^20^ Higher rates of bleeding have been seen in patients with higher Child‐Pugh and MELD scores.^1^, ^3^ Previous studies have also found a significant association between AKI and bleeding risk.^19^, ^20^, ^21^, ^22^

There is variability in the management of coagulopathy and antithrombotic agents for paracentesis.^23^ Cirrhotic patients have mixed coagulopathies where INR does not necessarily correlate to bleeding risk.^24^ If certain labs fall significantly out of generally agreed parameters (platelets <30 × 10^9 or INR > 2.5), thromboelastogram and fibrinogen measurements were used to quantify bleeding risk before the procedure.^25^ We also suspend chemical thromboprophylaxis and anticoagulation unless there is a strong indication otherwise.

Despite our individualized conservative approach, we reported a higher percentage of significant HGB drop following paracentesis, although only 0.49% of these had a hemorrhagic complication related to the procedure, which is in‐line with prior rates. The majority of the significant HGB declines that were not due to the paracentesis were multifactorial, including dilutional effects from IV fluid/albumin, bleeding from other sources (e.g., GIB), spurious values, etc. We did not investigate patients with nonsignificant HGB drops nor potential outcomes in patients after 48 h, therefore we may be underestimating patients who had minimal self‐resolved bleeding or delayed bleeding, although this is thought to be rare.

Our findings show a higher incidence of significant HGB drop in chronic viral hepatitis patients. There is no correlation between coagulopathic parameter, thrombocytopenia, use of antiplatelets or anticoagulation, multiple attempts, operator, renal dysfunction, or MELD. The best predictors of significant HGB drop appear to be AST, deeper ascites pocket, and LVP. It is unclear if elevated AST is truly related to an increased hemorrhagic risk. AST is present as cytosolic and mitochondrial isoenzymes and is found in organs outside the liver which perhaps suggests these patients are in a more acutely decompensated state, which places this group at higher risk of hemorrhage.^26^ Future studies could investigate this association further. Depth of pocket likely correlates with larger volume ascites and may lead to a larger decrease in intraabdominal pressure, leading to increased intraabdominal varix bleeding risk. However, this could also reflect a dilutional effect as the majority of patients receiving LVP receive albumin, and only one patient out of 45 with a significant HGB drop post LVP required a blood transfusion.

Although there was a perceived significant difference in death within 7 days of the procedure based on our initial inclusion criteria, further chart review confirmed that only 1.7% of deaths in the significant HGB drop group were related to the procedure, which was not statistically significant. Since no deaths in the nonsignificant HGB drop group were attributable to the procedure, overall mortality in the second cohort was 0.09%.

Interestingly, ascitic fluid RBC was higher in the significant HGB drop group, but the relevance of this is unclear. Future studies could investigate changes in the RBC count of ascites studies from the beginning to the end of the procedure to ascertain if any correlation with significant hemoglobin drop from hemoperitoneum. This could be potentially beneficial in patients deemed higher risk for bleeding. It also suggests that absence of bloody or blood‐tinged ascites (lower RBCs), does not lower the risk of the patient having a hemorrhagic complication.

## LIMITATIONS

This study has several limitations that warrant mentioning. While the study population was relatively large and heterogenous, there were limitations in the data collection, such as patients discharged postprocedure that led to a number of excluded patients. Additionally, there was a higher proportion of male patients in our cohort, which could limit generalization. The follow‐up period was 48 h postprocedure, leading to potential missed delayed complications such as delayed bleeding and AKI. We also did not investigate procedural bleeding in <2 g/dL drop in HGB. Another limitation to consider is the concomitant use of diuretics and its impact on our findings related to post‐paracentesis AKI.

## CONCLUSION

Bedside paracentesis still remains a relatively low‐risk procedure when utilizing ultrasound. However, when complications do occur, it is often associated with high morbidity. We found that post‐paracentesis AKI risk is not affected by the volume of ascites removed, but it is heightened in patients with higher MELD‐Na scores and when performed in patients already experiencing clinical deterioration. Additionally, the risk of hemorrhagic complications remained low in our cohort, but interestingly higher rates were seen in patients with elevated AST levels and those that had larger pockets of ascites pre‐procedure and larger volumes removed.

## CONFLICT OF INTEREST STATEMENT

The authors declare no conflicts of interest.

## Supporting information

Supporting information.

## References

[1] De Gottardi A , Thévenot T , Spahr L , et al. Risk of complications after abdominal paracentesis in cirrhotic patients: a prospective study. Clin Gastroenterol Hepatol. 2009;7(8):906‐909. 10.1016/j.cgh.2009.05.004 19447197

[2] Cho J , Jensen TP , Reierson K , et al. Recommendations on the use of ultrasound guidance for adult abdominal paracentesis: a position statement of the society of hospital medicine. J Hosp Med. 2019;14:E7‐E15. 10.12788/jhm.3095 30604780 PMC8021127

[3] Pache I , Bilodeau M . Severe, haemorrhage following abdominal paracentesis for ascites in patients with liver disease. Aliment Pharmacol Ther. 2005;21(5):525‐529. 10.1111/j.1365-2036.2005.02387.x 15740535

[4] Patel K , Tharalson E , Merill T , Gilani N . Large volume paracentesis can be performed safely by a nurse practitioner and can reduce physician work‐load in a busy GI practice: 335. Am J Gastroenterol. 2007;102:S238.

[5] Sharzehi K , Jain V , Naveed A , Schreibman I . Hemorrhagic complications of paracentesis: a systematic review of the literature. Gastroenterol Res Pract. 2014;2014:985141. 10.1155/2014/985141 25580114 PMC4280650

[6] Lin CH , Shih FY , Ma MHM , Chiang WC , Yang CW , Ko PCI . Should bleeding tendency deter abdominal paracentesis? Dig Liver Dis. 2005;37(Issue 12):946‐951. 10.1016/j.dld.2005.07.009 16185942

[7] Webster ST , Brown KL , Lucey MR , Nostrant TT . Hemorrhagic complications of large volume abdominal paracentesis. Am J Gastroenterol. 1996;91:366‐368. 8607508

[8] Arnold C , Haag K , Blum H , Rossle M . Acute hemoperitoneum after large‐volume paracentesis. Gastroenterology. 1997;113:978‐982. 10.1016/S0016-5085(97)70210-5 9287992

[9] Barsuk JH , Rosen BT , Cohen ER , Feinglass J , Ault MJ . Vascular ultrasonography: a novel method to reduce paracentesis related major bleeding. J Hosp Med. 2018;13(1):30‐33. 10.12788/jhm.2863 29073312

[10] Harris PA , Taylor R , Thielke R , Payne J , Gonzalez N , Conde JG . Research electronic data capture (REDCap) – a metadata‐driven methodology and workflow process for providing translational research informatics support. J Biomed Inf. 2009;42(2):377‐381. 10.1016/j.jbi.2008.08.010 PMC2700030 18929686

[11] Harris PA , Taylor R , Minor BL , et al. The REDCap consortium: building an international community of software partners. J Biomed Inform. 2019;95:103208. 31078660 10.1016/j.jbi.2019.103208 PMC7254481

[12] Tanaka T , Vander Weg M , Jones M , Wehby G . Assessment of the 2021 AASLD practice guidance for albumin infusion in elective therapeutic paracentesis: a regression discontinuity design. Am J Gastroenterol. 2024;119:2045‐2051. 38501671 10.14309/ajg.0000000000002767 PMC13575889

[13] Aithal GP , Palaniyappan N , China L , et al. Guidelines on the management of ascites in cirrhosis. Gut. 2021;70(1):9‐29. 33067334 10.1136/gutjnl-2020-321790 PMC7788190

[14] Bernardi M , Caraceni P , Navickis RJ , Wilkes MM . Albumin infusion in patients undergoing large‐volume paracentesis: a meta‐analysis of randomized trials. Hepatology. 2012;55(4):1172‐1181. 10.1002/hep.24786 22095893

[15] Patil V , Jain M , Venkataraman J . Paracentesis‐induced acute kidney injury in decompensated cirrhosis ‐ prevalence and predictors. Clin Exp Hepatol. 2019;5(1):55‐59. 10.5114/ceh.2019.83157 30915407 PMC6431093

[16] Seethapathy H , Sharma S , Zhao S , et al. Acute kidney injury following paracentesis among inpatients with cirrhosis. Kidney Int Rep. 2020;5(8):1305‐1308. 10.1016/j.ekir.2020.05.014 32775831 PMC7403547

[17] Thomson A , Cain P , Kerlin P , Strong R . Serious hemorrhage complicating diagnostic abdominal paracentesis. J Clin Gastroenterol. 1998;26(4):306‐308. 10.1097/00004836-199806000-00020 9649018

[18] Mallory A . Complications of diagnostic paracentesis In patients with liver disease. JAMA: J Am Med Assoc. 1978;239(7):628‐630. 10.1001/jama.1978.03280340048020 146097

[19] McVay PA , Toy PTCY . Lack of increased bleeding after paracentesis and thoracentesis in patients with mild coagulation abnormalities. Transfusion. 1991;31(2):164‐171. 10.1046/j.1537-2995.1991.31291142949.x 1996485

[20] Hung A , Garcia‐Tsao G . Acute kidney injury, but not sepsis, is associated with higher procedure‐related bleeding in patients with decompensated cirrhosis. Liver Int. 2018;38(8):1437‐1441. 10.1111/liv.13712 29393567 PMC6072624

[21] Zanetto A , Rinder HM , Campello E , et al. Acute kidney injury in decompensated cirrhosis is associated with both hypo‐coagulable and hyper‐coagulable features. Hepatology. 2020;72(4):1327‐1340. 10.1002/hep.31443 32614088 PMC8672302

[22] Zarka F , Tayler‐Gomez A , Ducruet T , et al. Risk of incident bleeding after acute kidney injury: a retrospective cohort study. J Crit Care. 2020;59:23‐31. 10.1016/j.jcrc.2020.05.003 32485439

[23] Blank JA , Peters KK , O'Donnell MA , Mansoor AM . Clinical progress note: consolidated guidelines on management of coagulopathy and antithrombotic agents for common bedside procedures. J Hosp Med. 2021;16:675‐679. 10.12788/jhm.3700 34730498

[24] Munoz SJ , Stravitz RT , Gabriel DA . Coagulopathy of acute liver failure. Clin Liver Dis. 2009;13(1):95‐107. 10.1016/j.cld.2008.10.001 19150314

[25] Stravitz RT . Potential applications of thromboelastography in patients with acute and chronic liver disease. Gastroenterol Hepatol. 2012;8(8):513‐520. PMC3533209 23293564

[26] Lee TH , Kim WR , Poterucha JJ . Evaluation of elevated liver enzymes. Clin Liver Dis. 2012;16(2):183‐198. 10.1016/j.cld.2012.03.006 22541694 PMC7110573

