# Supplementary material for: Paracentesis outcomes from a medicine procedure service at a tertiary care transplant center
Source: J Hosp Med. 2025 Mar 27;20(9):943–52. doi: 10.1002/jhm.70037 (PMC12406758; doi:10.1002/jhm.70037)
Supplement: Supplementary file 1 — Supporting information. [file JHM-20-943-s001.pdf]

Procedure Medicine Data

Please complete the form below for each consult.

Thank you!

Patient Name

____
(e.g. John Doe)

MRN

____

Age

____

Sex

- ◯ Male
- ◯ Female

Ethnicity

- ◯ Black
- ◯ White
- ◯ Hispanic
- ◯ Asian
- ◯ Other

BMI

____

Procedure

- ◯ Paracentesis
- ◯ Lumbar Puncture
- ◯ Central Line
- ◯ Temporary HD Line
- ◯ Peripheral IV
- ◯ Arthrocentesis
- ◯ Other

"Other" Procedure

____

Date Consulted

____

Requesting Team

- ◯ Dig health
- ◯ Team 1
- ◯ Team 2
- ◯ Team 3
- ◯ Team 4
- ◯ Team 5
- ◯ HMS
- ◯ Heme
- ◯ Onc
- ◯ ACE (Cardiology)
- ◯ Neuro
- ◯ ED
- ◯ CDU
- ◯ Ortho
- ◯ Palliative
- ◯ Psychiatry
- ◯ OBGYN
- ◯ EGS/Trauma
- ◯ Transplant surgery
- ◯ Urology

	◯ Inpatient rehab<br>◯ Surg onc<br>◯ Neurosurgery<br>◯ Plastics<br>◯ ENT<br>◯ BMT<br>◯ Other
"Other" Requesting Team	__________________________
Consult Performed?	◯ Yes<br>◯ No
Procedure Attempted?	◯ Yes<br>◯ No
Why was procedure not attempted?	◯ Patient declined<br>◯ Not indicated<br>◯ Contraindication<br>◯ Concern for ability to perform safely<br>◯ Request cancelled by team<br>◯ Able to obtain other vascular access<br>◯ Other
Date Attempted	__________________________
Hospital floor where procedure performed	◯ 8W<br>◯ 9C<br>◯ 9E<br>◯ 9W<br>◯ Main 10<br>◯ 11E<br>◯ 11 EMU<br>◯ 11W<br>◯ CSBC<br>◯ CCH2<br>◯ CCH3<br>◯ CCH7<br>◯ CDU<br>◯ Emergency department<br>◯ N4<br>◯ N5<br>◯ N6<br>◯ N9<br>◯ N10<br>◯ Other<br>◯ CCH9<br>◯ PACU<br>◯ N1<br>◯ N2
"Other" Hospital Floor	__________________________
Indication for paracentesis	☐ New onset ascites<br>☐ Clinical deterioration of patient with ascites<br>☐ Management of tense/diuretic resistant ascites<br>☐ Other
"Other" paracentesis indication	__________________________

Etiology of ascites?	☐ Chronic Viral Hepatitis<br>☐ Alcoholic Liver Disease<br>☐ Nonalcoholic Fatty Liver Disease<br>☐ Cardiogenic<br>☐ Autoimmune<br>☐ Malignant<br>☐ Unknown<br>☐ Other
Diagnostic paracentesis?	○ Yes<br>○ No
Therapeutic paracentesis?	○ Yes<br>○ No
Serum Na	__________________________
AST	__________________________
ALT	__________________________
T Bili	__________________________
Renal Function	☐ Normal<br>☐ AKI<br>☐ CKD<br>☐ ESRD<br>(Mark AKI if increase in Cr >0.3 from baseline within 24 hours or >=50% increase using the last available value of SCr within last 3 months)
Receiving Dialysis (HD or CRRT)?	○ Yes<br>○ No
Serum creatinine prior to paracentesis (within 72 hours)	__________________________
Serum creatinine after paracentesis	__________________________
Hemoglobin prior to procedure	__________________________
Hemoglobin post procedure (use AM if collected)	__________________________
Did the patient receive a blood transfusion post procedure?	○ Yes<br>○ No<br>(Mark yes if received a transfusion within 48 hours post-procedure)
Platelets (within 72 hours)	__________________________
PT/INR (within 72 hours)	

	______________________________
PTT (within 72 hours)	______________________________
Anticoagulation?	○ No<br>○ Prophylactic<br>○ Therapeutic
Anticoagulation held for procedure?	○ Yes<br>○ No
What medication?	______________________________
Site of paracentesis	○ Left lower quadrant<br>○ Right lower quadrant<br>○ Other
Depth of pocket seen on US for paracentesis (in cm)	______________________________<br>(Required if therapeutic paracentesis)
Amount of fluid removed during paracentesis (in liters)	______________________________
Ascites RBC count (x 10^6L)	______________________________
Ascites total nucleated count (x 10^6L)	______________________________
Ascites % Poly	______________________________
Culture positive?	○ Yes<br>○ No
Organism isolated?	☐ Gram Positive<br>☐ Gram Negative<br>☐ Fungus<br>☐ No organism isolated<br>☐ Other
Antibiotics at time of procedure?	○ Yes<br>○ No
What antibiotics?	☐ Beta lactam<br>☐ Fluoroquinolones<br>☐ Zosyn/Augmentin<br>☐ Vancomycin<br>☐ Clindamycin<br>☐ Daptomycin<br>☐ Macrolide<br>☐ Tetracycline<br>☐ Carbapenem<br>☐ Metronidazole<br>☐ Linezolid

☐ Antifungal
☐ Other

Performed by

____
(e.g. John Doe)

Supervisor

○ [illegible]
○ [illegible]
○ [illegible]
○ [illegible]
○ [illegible]
○ [illegible]
○ [illegible]
○ [illegible]
○ [illegible]
○ [illegible]
○ [illegible]
○ [illegible]
(e.g. John Doe)

Number of attempts

____

Procedure completed?

○ Yes
○ No

Why was the procedure not completed?

○ Aborted (e.g. patient related factors)
○ Failed (e.g. equipment failure, anatomy)

Comments about why the procedure was not completed?

____

Referred to IR?

○ Yes
○ No

Reason for referral to IR?

____

Immediate complications?

○ Yes
○ No

What immediate complications occurred?

____

Delayed complications?

○ Yes
○ No
(Next day follow-up)

What delayed complications occurred?

____

Facilitated throughput?

☐ Prevented admission
☐ Procedure performed pre-discharge
☐ Completed on a weekend

Has the patient been discharged post-paracentesis?

○ Yes
○ No

Date of admission?	______________________
Date of discharge?	______________________
Death this admission?	◯ Yes<br>◯ No
Was death related to the procedure?	◯ Yes<br>◯ No
Any comments?	______________________<br>(e.g. equipment issues, staffing, or etc.)
